# The Efficacy of Dienogest in Reducing Disease and Pain Recurrence After Endometriosis Surgery: a Systematic Review and Meta-Analysis

**DOI:** 10.1007/s43032-023-01266-0

**Published:** 2023-05-22

**Authors:** Ludovico Muzii, Chiara Di Tucci, Giulia Galati, Fabiana Carbone, Innocenza Palaia, Giorgio Bogani, Giorgia Perniola, Federica Tomao, Evangelos Kontopantelis, Violante Di Donato

**Affiliations:** 1 Department of Maternal Infantile and Urological Sciences, Sapienza University of Rome, Viale del Policlinico, 155-00161 Rome, Italy; 2 Division of Informatics, Imaging and Data Sciences, University of Manchester, Greater Manchester, Manchester, UK

**Keywords:** Dienogest, Endometriosis, Endometrioma, Recurrence, Postoperative medical treatment

## Abstract

The objective of this study is to determine whether dienogest therapy after endometriosis surgery reduces the risk of recurrence compared with placebo or alternative treatments (GnRH agonist, other progestins, and estro-progestins). The design used in this study is systematic review with meta-analysis. The data source includes PubMed and EMBASE searched up to March 2022. A systematic review and meta-analysis were performed in accordance with guidelines from the Cochrane Collaboration. Keywords such as “dienogest,” “endometriosis surgery,” “endometriosis treatment,” and “endometriosis medical therapy” were used to identify relevant studies. The primary outcome was recurrence of endometriosis after surgery. The secondary outcome was pain recurrence. An additional analysis focused on comparing side effects between groups. Nine studies were eligible, including a total of 1668 patients. At primary analysis, dienogest significantly reduced the rate of cyst recurrence compared with placebo (*p* < 0.0001). In 191 patients, the rate of cyst recurrence comparing dienogest vs GnRHa was evaluated, but no statistically significant difference was reported. In the secondary analysis, a trend toward reduction of pain at 6 months was reported in patients treated with dienogest over placebo, with each study reporting a significantly higher reduction of pain after dienogest treatment. In terms of side effects, dienogest treatment compared with GnRHa significantly increased the rate of spotting (*p* = 0.0007) and weight gain (*p* = 0.03), but it was associated with a lower rate of hot flashes (*p* = 0.0006) and a trend to lower incidence of vaginal dryness. Dienogest is superior to placebo and similar to GnRHa in decreasing rate of recurrence after endometriosis surgery. A significantly higher reduction of pain after dienogest compared with placebo was reported in two separate studies, whereas a trend toward reduction of pain at 6 months was evident at meta-analysis. Dienogest treatment compared with GnRHa was associated with a lower rate of hot flashes and a trend to lower incidence of vaginal dryness.

**Supplementary Information:**

The online version contains supplementary material available at 10.1007/s43032-023-01266-0.

## Introduction

Endometriosis is an estrogen-dependent, chronic disease characterized by the presence of endometrial glands and stroma outside the uterus. Endometriotic disease affects about 5% of women of reproductive age [1] and is frequently associated with pelvic pain and/or infertility [2]. Ovarian endometriomas are present in up to 41% of patients with endometriosis [3, 4], whereas deeply infiltrating endometriosis (DIE) has been reported in 39% of the cases of pelvic endometriosis [5]. Management options in case of endometriosis include medical therapy, surgery, assisted reproductive techniques (ART) in case of associated infertility, or a combination of the above [3, 5–7]. Among the available medical therapies, combined oral contraceptives (COC) and progestins are usually considered first-line options [6, 7].

Dienogest (DNG) is a fourth-generation progestin that has been approved for the medical treatment of endometriosis and has the advantage of having little androgenic, glucocorticoid, or mineralocorticoid properties. Nowadays, only few controlled trials evaluating the effect of dienogest compared to placebo or other medical treatments in patients affected by endometriosis have been published. We deemed it relevant to conduct a systematic literature review and meta-analysis with the objective of defining the magnitude of the effect of dienogest in reducing lesion and symptom recurrence after conservative surgery for endometriosis and comparing the impact of dienogest with that of GnRH agonists and other medical treatments when used as a postoperative preventive measure.

## Materials and Methods

### Search Strategy

The present systematic review and meta-analysis were performed in accordance with guidelines from the Cochrane Collaboration and followed Preferred Reporting Items for Systematic Reviews and Meta-Analyses guidelines [8]. The study protocol was registered online in the International Prospective Register of Systematic Reviews (PROSPERO number: CRD3274812022).

An electronic database search was performed to identify articles published until March 2022. PubMed and EMBASE were screened to identify studies that evaluated the efficacy of dienogest in the management of endometriosis after surgery, using a combination of the following search terms: “dienogest,” “endometriosis surgery,” “endometriosis treatment,” and “endometriosis medical therapy.”

A broadly inclusive search was conducted initially, followed by a subsequent restriction for studies on patients undergoing surgery during the title/abstract review process.

In the attempt to identify further published, unpublished, and ongoing trials, we searched trials and research registries (ClinicalTrials.gov, australianclinicaltrials.gov.au). The reference lists of reviews and relevant articles were screened by hand to identify additional eligible publications. The search strategy is described in detail in Supplementary Data File S1, available online. Articles considered were randomized clinical trials (RCTs), prospective, or retrospective controlled studies evaluating the effect of dienogest compared to placebo/no therapy or other treatment (GnRH agonist, other progestins, or combined oral contraceptives) to prevent the recurrence of endometriosis after surgery. The protocol was designed a priori, defining methods for collecting, extracting, and analyzing data.

The electronic search was conducted independently by two investigators (G.G. and F.C.). All articles considered relevant based on the title and abstract were retrieved. Subsequently, five investigators (L.M., C.D.T., V.D.D., G.G., and F.C.) independently read the full text of the pre-selected articles to verify the pertinence of the articles for the aim of the analysis. Studies were excluded if reporting partial or duplicate data sets. In case of disagreement on the inclusion or exclusion of preselected studies for meta-analysis or any other disagreement through the review process, the consensus was reached after discussion involving all researchers.

### Inclusion Criteria

Controlled studies (retrospective or prospective) evaluating the risk of disease recurrence and changes in endometriosis-related pain in premenopausal women undergoing endometriosis curative surgery followed by dienogest vs placebo/no therapy, or other hormonal suppression were included.

**Fig. 1:**
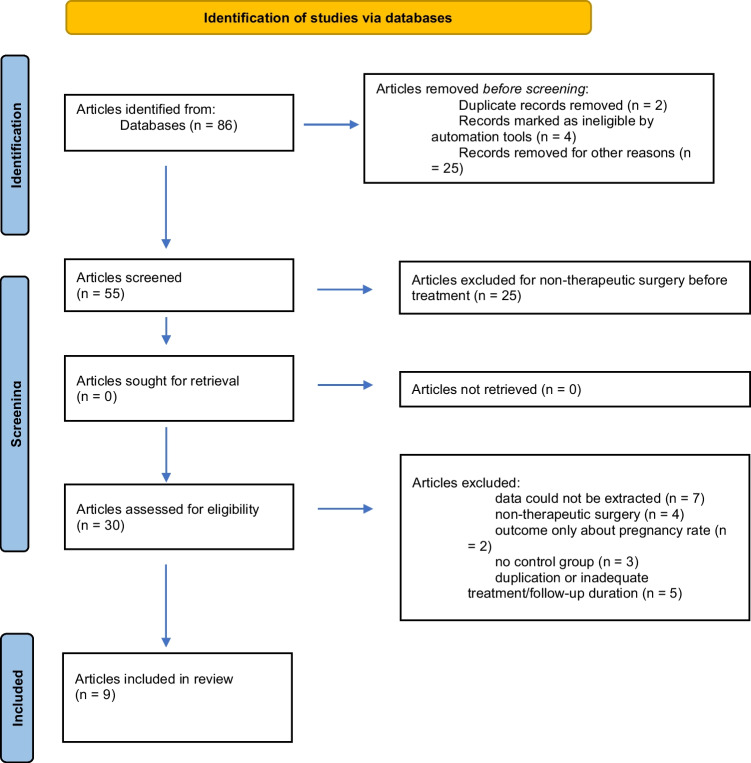
PRISMA flow chart for study identification and inclusion/exclusion

Inclusion criteria were (1) English language, (2) presence of a control group, and (3) evaluation of at least one outcome of interest. After confirmation of pertinence, studies were excluded if they report partial or incomplete data. Studies evaluating patients without histologically proven endometriosis and those who underwent only diagnostic, non-curative surgery were excluded from our analysis. The same subjects were not included twice in an analysis of a single outcome. A flow diagram of the study selection process is presented in Fig. 1.

### Quality Assessment

All identified controlled studies were included in the meta-analysis. The studies were then classified qualitatively according to the guidelines published in the Cochrane Handbook for Systematic Reviews of Interventions. The Risk of Bias in Non-randomized Studies of Interventions (ROBINS-I) tool was used for assessing the risk of bias in non-randomized studies of interventions included in the meta-analysis (Supplemental Table 1).

### Outcomes

Outcomes that are particularly concerning for patients in this context were selected.

#### Primary Outcome

1. Recurrence rate: Evaluation of the postoperative recurrence rate of endometriosis, defined as imaging evidence of a new endometrioma of more than 15 mm, plaques, and/or endometriosis nodules either on ultrasound (US) or magnetic resonance imaging (MRI).

#### Secondary Outcomes

2) Pelvic pain recurrence: Evaluation of pelvic pain using standardized measures (10-point Visual Analogue Scale, with conversion to a 10-point scale in case studies reported a 1–100 mm scale) to score the symptom intensity from preoperative baseline to follow-up period.
3) Side effects: Evaluation of side effects according to the drug used.
  The occurrence of spotting, depression, headache, vaginal dryness, weight gain, and hot flashes from baseline have been extrapolated from the studies for each side effect.

### Analysis

Data were pooled using RevMan software (Review Manager version 5.4; the Cochrane Collaboration, Copenhagen, Denmark). Dichotomous outcomes from each study were expressed as odds ratios (OR) with a 95% confidence interval (CI). Continuous outcomes were expressed as standardized mean differences (SMD). Heterogeneity between studies was reported with the *I*^2 statistic. A DerSimonian-Laird random-effects meta-analysis model was used at meta-analysis if any heterogeneity was detected, whereas a fixed-effects model was used if no heterogeneity was identified. A value of *p* < 0.05 was considered statistically significant. We decided to examine publication bias with Egger’s test and funnel plots if the number of studies was 10 or above because these analyses are underpowered otherwise [8].

## Results

### Study Selection

Our electronic database search produced 86 articles. Title and abstract screening selected a total of 30 studies eligible for full-text evaluation. A total of 21 of these papers were excluded, as detailed in the PRISMA flowchart in Fig. 1 and Supplemental Table 2, available online. Nine studies fulfilled the inclusion criteria: four were prospective controlled studies and five were retrospective studies. Recurrence rate was reported in six studies [9–14]. Change in pelvic pain was reported in three studies [9, 13, 15]. Side effects were reported in four studies (13–16).

### Study Characteristics

Nine studies were included in the meta-analysis, encompassing a total of 1668 patients. Globally, 581 (9 studies) women received, after surgery for endometriosis, dienogest and 345 other medical treatments, of which 281 (3 studies) were GnRH analogs (GnRHa), 64 (5 studies) were estroprogestins, and 742 (6 studies) were placebo or no therapy.

### Effects of Interventions

#### Recurrence rate

##### **Dienogest vs Placebo/No Therapy**

**Fig. 2:**
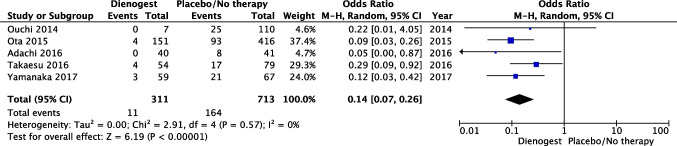
Forest plot: endometriosis recurrence with post-operative dienogest compared to placebo/no therapy

Five articles with a total of 1024 patients (311 in the DNG group and 713 in the placebo group) evaluated the rate of cyst recurrence comparing DNG vs placebo/no therapy during the follow-up period ranging from 24 to 60 months (9–13). Definitions of recurrence reported in trials included in the meta-analysis are reported in Table 1. In particular, two studies defined recurrence as the evidence of a new endometrioma of more than 2 cm on ultrasound (US) [9, 10], two magnetic resonance imaging (MRI) [12, 13], and one the presence of endometrioma with a minimum diameter of 15 mm based on noninvasive imaging [11]. Dienogest significantly reduced the rate of cyst recurrence compared with placebo or no treatment. The pooled estimated odds ratio (OR) was 0.14 (95% CI 0.07 to 0.26; *p* < 0.0001). Heterogeneity for this comparison was I2 0% (95% CI 0–79.2%). Only one study was prospective, making any statistical subgroup analysis according to the study design (prospective vs retrospective) impractical (Fig. 2).

**Table 1:** General characteristics of the included studies

Author and year	Study design	Patient numbers	Age, mean	Intervention	Control group
Ouchi et al. 2014	RS	7	34.6 ± 5.8	DNG 2 mg/day, orally	1. No therapy
					2. Continuous COC, orally
					3. Discontinued COC, orally
					4. LA 1.88 mg or buserelin 1.8 mg/4 week, subcutaneously
Ota et al. 2015	RS	151	32.56 ± 5.23	DNG 2 mg/day, orally	No therapy
Adachi et al. 2016	RS	40	35.4 ± 1.0	DNG 2 mg/day, orally	No therapy
Lee et al. 2016	PS	36	29.0 ± 5.9	DNG 2 mg/day, orally	LA 3.75 mg/4 week, subcutaneously and add–back
Takaesu et al. 2016	PRT	56	34.1 ± 6.6	DNG 2 mg/day, orally	1. No therapy
					2. Goserelin 1.8 mg/4 week, subcutaneously
Yamanaka et al. 2017	RS	59	35 ± 6.8	DNG 2 mg/day, orally	No therapy
Abdou et al. 2018	PRT	121	29.52 ± 3.32	DNG 2 mg/day, orally	LA 3.75 mg/4 weeks, intramuscularly
Ceccaroni et al. 2021	PRT	81	35 ± 5.5	DNG 2 mg/day, orally	Triptorelin or leuprorelin 3.75mg /4 weeks, intramuscularly
Kashi et al. 2021	PRT	30	34.22 ± 6.54	DNG 2 mg/day, orally	1. Placebo
					2. COC, orally

Abbreviations: *COC*, combined oral contraceptive; *DNG*, dienogest; *LA*, leuprolide acetate; *PS*, prospective study; *PRT*, prospective randomized study; *RS*, retrospective study

##### **Dienogest vs GnRHa**

**Fig. 3:**
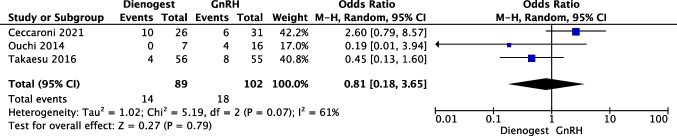
Forest plot: endometriosis recurrence with post-operative dienogest compared to GnRHa

Three articles [10, 13, 14] with a total of 191 patients (89 in the dienogest group and 102 in the GnRHa group) evaluated the rate of cyst recurrence comparing DNG vs GnRHa during the follow-up period ranging from 24 to 60 months. Heterogeneity for this comparison was I2 61% (95% CI 0–88.9%). No statistically significant difference was reported between groups (OR 0.81; 95% CI 0.18–3.65; *p* = 0.79) (Fig. 3).

##### **Dienogest vs Other Progestins**

No study reports any data about this outcome.

##### **Dienogest vs Estroprogestins**

Only one monocentric [10] retrospective study evaluated the recurrence comparing dienogest (*n* = 7) vs continuous (*n* = 25) or cyclic (*n* = 9) oral contraceptive pill. That study reported no recurrence in DNG and continuous contraceptive groups, whereas there was 5 (55%) in cyclic oral contraceptive group 5 years after surgery.

#### Pelvic Pain

##### **Dienogest vs Placebo/No Therapy**

**Fig. 4:**
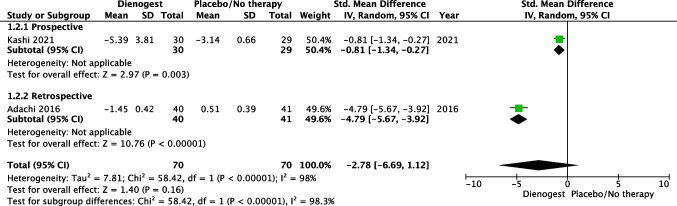
Forest plot: changes in pelvic pain at 6 months comparing dienogest vs placebo/no therapy

Two studies with a total of 140 patients (70 in the dienogest group and 70 in the placebo group) evaluated changes in pelvic pain at 6 months comparing DNG vs placebo/no therapy [9, 17]. Heterogeneity for this comparison was I2 98% (95% CI 95.3–99.1%). The standard mean difference (SMD) for pain at baseline vs 6 months reported on a 10-point scale was − 2.78 (95% CI − 6.69 to 1.12), *p* = 0.16 (Fig. 4). Only one study comparing DNG vs placebo/no therapy evaluated changes in pelvic pain at 12 and 24 months [9]. This study suggests that DNG significantly reduced pain compared with placebo at 12 (SMD: − 4.31; 95% CI − 5.29 to 3.33; *p* < 0.0001) and 24 months (SMD: − 3.50; 95% CI − 4.71 to 2.28; *p* < 0.0001).

##### **Dienogest vs GnRHa**

Only one study evaluated changes in pelvic pain at 3 months comparing DNG vs GnRHa, and no difference was observed [15].

##### **Dienogest vs Progestins**

No study reports any data about this outcome.

##### **Dienogest vs Estroprogestins**

Only one monocentric randomized, double-blind, placebo-controlled study [17] evaluated pelvic pain by comparing DNG (*n* = 30) vs continuous oral contraceptive pill (*n* = 30). That study reported the mean difference and no significant difference was registered between the two intervention groups.

### Adverse Effects

#### Dienogest vs Placebo/No Therapy

All studies reported adverse events only in the treatment group and therefore were not considered in the meta-analysis.

##### **Dienogest vs GnRHa**

**Spotting**

**Fig. 5:**
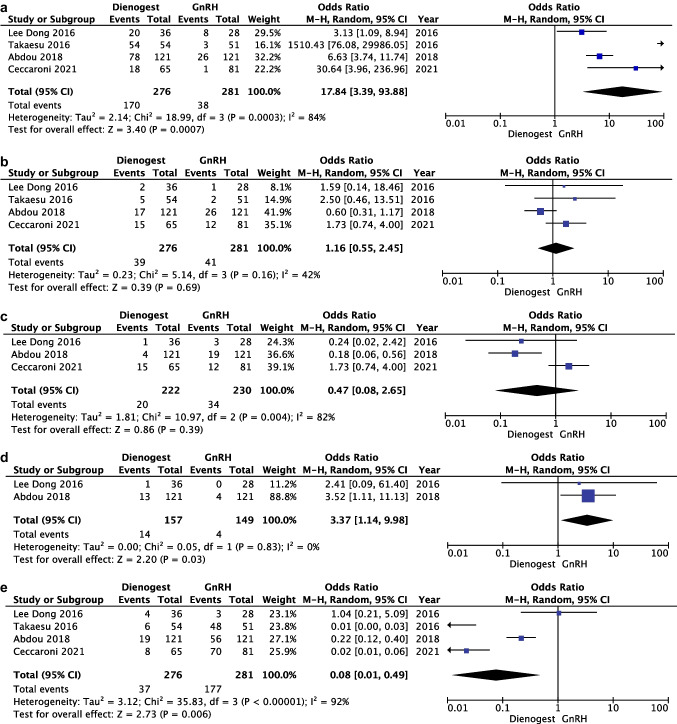
**a** Forest plot: rate of spotting comparing dienogest vs GnRHa. **b** Forest plot: rate of headache comparing dienogest vs GnRHa. **c** Forest plot: rate of vaginal dryness comparing dienogest vs GnRHa. **d** Forest plot: rate of weight gain comparing dienogest vs GnRHa. **e** Forest plot: rate of hot flashes comparing dienogest vs GnRHa

Four articles with a total of 557 patients (276 in the dienogest group and 281 in the GnRHa group) evaluated the rate of spotting comparing DNG vs GnRHa (13–16). Heterogeneity for this comparison was I2 84% (95% CI 96.7–98.8%). A higher rate of spotting was reported in dienogest group compared with GnRHa group. The pooled estimated odds ratio (OR) was 17.84 (95% CI 3.39 to 93.88; *p* = 0.0007) (Fig. 5a).

**Depression**

Only one study reported data on depression [16]. That study suggests that after 6 months, DNG vs GnRHa are similar in terms of incidence of depression.

**Headache**

Four articles with a total of 557 patients (276 in the dienogest group and 281 in the GnRHa group) evaluated the rate of headache comparing DNG vs GnRHa (13–16). Heterogeneity for this comparison was I2 42% (95% CI 0–80.5%). The pooled estimated OR was 1.16 (95% CI 0.55–2.45); *p* = 0.69 (Fig. 5b).

**Vaginal Dryness**

Three articles with a total of 452 patients (222 in the dienogest group and 230 in the GnRHa group) evaluated the rate of vaginal dryness comparing DNG vs GnRHa (14–16). Heterogeneity for this comparison was I2 82% (95% CI 44.4–94.2%). A trend of higher incidence of vaginal dryness was reported in the GnRHa group. The pooled estimated OR was 0.47 (95% CI 0.08–2.65); *p* = 0.39 (Fig. 5c).

**Weight Gain**

Two articles with a total of 306 patients (157 in the dienogest group and 149 in the GnRHa group) evaluated the rate of weight gain comparing DNG vs GnRHa [15, 16]. A higher rate of weight gain was reported in the DNG group compared with the GnRHa group. Heterogeneity for this comparison was I2 0% (95% CI 0–98.2%). The pooled estimated odds ratio (OR) was 3.37 (95% CI 1.14 to 9.98; *p* = 0.03) (Fig. 5d).

**Hot Flashes**

Four articles with a total of 557 patients (276 in the dienogest group and 281 in the GnRHa group) evaluated the rate of hot flashes comparing DNG vs GnRHa (13–16). Heterogeneity for this comparison was I2 92% (95% CI 95.7–98.3%). A lower rate of hot flashes was reported in the DNG group compared with the GnRHa group. The pooled estimated odds ratio (OR) was 0.08 (95% CI 0.01 to 0.49; *p* = 0.006) (Fig. 5e).

## Discussion

The present meta-analysis summarizes the highest-quality evidence available in English-language gynecology literature on the efficacy of DNG for medical treatment of endometriosis after surgical excision of the disease. On the basis of the analyzed outcomes, DNG is superior to placebo or no treatment and similar to GnRHa in decreasing rate of recurrences after conservative surgery for treatment of endometriosis. In the present meta-analysis, only one monocentric [10] retrospective study evaluated the rate of recurrence after surgery for endometriosis, comparing dienogest vs continuous or cyclic oral contraceptive pill. This study reports a statistically higher rate of recurrence in patients who underwent postoperative cyclic compared to continuous oral contraceptives or dienogest. Moreover, as results from analysis of secondary outcome, a trend toward reduction of 6 months pain was reported (SMD – 2.78; 95% CI − 6.69 to 1.12; *p* = 0.16) in patients treated with DNG over placebo or no therapy. This finding could be influenced by the availability in the literature of only 2 small and very heterogeneous studies. Interestingly, both of them reported a significantly higher reduction of pain after DNG compared with placebo or no therapy (DNG: − 5.39 ± 3.81 vs no therapy − 3.14 ± 0.66, *p* < 0.05 [17] and DNG: − 1.45 ± 0.42 vs no therapy 0.51 ± 0.39, *p* = 0.0013 (9). Furthermore, one of them suggested a significantly reduced pain with DNG over placebo also at 12 and 24 months (9). Only one study [15] evaluated changes in pelvic pain at 3 months, comparing dienogest vs GnRHa showing no difference. Considering drug-related side effects, the present meta-analysis showed that, if, on one hand, dienogest treatment compared with GnRHa significantly increased the rate of spotting occurrence (OR: 17.84; *p* = 0.0007) and weight gain (OR: 3.37; *p* = 0.03), on the other hand, it is associated with a lower rate of hot flashes (OR: 0.08; *p* = 0.0006) and a trend toward a lower incidence of vaginal dryness (OR: 0.61; *p* = 0.10).

Endometriosis tends to recur after surgery in as many as 89.6% of cases (6). Endometrioma recurrence may be prevented with postoperative long-term (> 12 months) hormonal treatment (6). In a systematic review by Chen et al. [19], a reduction of disease recurrence in favor of postsurgical hormonal therapy (combined oral contraceptives, GnRHa, and danazol) was reported compared to no postsurgical hormonal therapy (RR: 0.40; 95% CI: 0.27 to 0.58).

In a systematic review by Zakhri et al. [20], dienogest proved superior to expectant management with respect to endometriosis recurrence after surgery (2% vs 29%; log odds − 1.96, 95%CI: − 2.53 ± 1.38). No comparison was made in this review with other medical therapies.

In a second systematic review by Zakhri et al. [21], a pooled analysis of postoperative medical therapies showed a reduction in disease recurrence compared to postoperative medical therapies (RR: 0.41; 95%CI: 0.26 ± 0.65). In the above review, the subgroup analysis for each hormonal therapy (combined hormonal contraceptives, progestins, androgens, levonorgestrel-releasing intra-uterine system, or GnRH agonist or antagonist) showed no significant difference for progestin therapy since only a single study was included.

In a systematic review by Liu et al. [22] dienogest treatment after surgery was compared to no treatment (4 studies) or other treatments (GnRH-a, 5 studies; LNG-IUS, 2 studies). Dienogest therapy was associated with a lower rate of disease recurrence (OR: 0.14; 95%CI: 0.07 ± 0.26 vs no treatment, and OR 0.46; 95%CI: 0.24 ± 0.86 vs other treatments).

The above and the present study therefore consistently report that postoperative disease recurrence may be reduced with long-term medical therapy. Postoperative medical therapy should be suggested whenever the patient is not seeking a conception and should be continued indefinitely until pregnancy is desired. In the present study, dienogest proved superior to both no treatment and other medical therapies. Given the favorable side effects and cost profiles of the progestins compared to other classes of medical therapies, dienogest may be considered among the first-line options for the prevention of endometrioma recurrence after surgical excision (6). As an example of costs, 1-month course of therapy with triptorelin in Italy will cost 171 €, compared to 15 € for a combined oral contraceptive (2 mg of dienogest and 30 μg of ethinyl estradiol) and 17 € for 2 mg of dienogest [23].

The present analysis has some limitations. First, randomized trials are few in number and, in some cases, of poor quality. Second, the definition of recurrence and follow-up are heterogeneous, and the analysis of both randomized and non-randomized studies could lead to selection and information bias. Third, the results of the present meta-analysis are applicable only to patients undergoing surgical treatment of endometriomas and cannot be generalizable to other phenotypes of disease, i.e., DIE and superficial endometriosis. Finally, an additional potential limitation is the heterogeneity level that often remains undetected in small meta-analyses, therefore leading to imprecise pooled estimates [18]. However, in most of present meta-analysis, heterogeneity was successfully modeled using random-effects meta-analysis methods. Awaiting more consolidated data on these specific outcomes, it should be acknowledged that the present meta-analysis has several strengths: it represents a comprehensive evaluation with a good quality of methodological assessment and strict inclusion criteria of all currently available data on dienogest after surgical therapy, providing a large sample size, the quality of the methodology assessment, and strict inclusion criteria. Combined information on benefit and potential side effects of dienogest compared with other treatment strategies are really useful, as they help physicians to adequately counsel patients on choosing the best personalized treatment.

In conclusion, dienogest appears as a safe and more effective method of prevention of postoperative disease and pain recurrence after surgery for endometriosis [24, 25] compared to placebo or no treatment. DNG is as effective as GnRHa for cyst and pain recurrence with less severe side effects, particularly for bothersome side effects such as hot flashes and vaginal dryness. On the other hand, DNG is associated with higher rates of spotting, which however are better tolerated by patients, especially when correctly informed.

### Supplementary Information

ESM 1

ESM 2

ESM 3

## Data Availability

Data regarding any of the subjects in the study has not been previously published unless specified. Data will be made available to the editors of the journal for review or query upon request.
