# Supplementary material for: The Efficacy of Dienogest in Reducing Disease and Pain Recurrence After Endometriosis Surgery: a Systematic Review and Meta-Analysis
Source: Reprod Sci. 2023 May 22;30(11):3135–43. doi: 10.1007/s43032-023-01266-0 (PMC10643411; doi:10.1007/s43032-023-01266-0)
Supplement: Supplementary file 1 — ESM 1 [file 43032_2023_1266_MOESM1_ESM.docx]

**Supplementary Data File 1 – Search Methods (Pubmed/Medline Database)**

A comprehensive search approach was used to locate published studies.

All databases were searched since January 2012 to March 2022.

1. dienogest: “dienogest” OR “Visanne” (613 results)

2. endometrioma: “endometrioma” OR “endometriomas” OR “chocolate cyst” OR “ovarian endometrioma” OR “endometriotic cyst” OR “endometriosis”(31,753 results)

3. medical therapy: “medical treatment” OR “medical therapy” (79,886 results)

4. endometriosis recurrence: “endometriosis recurrence” OR “endometrioma recurrence” OR “endometriomas recurrence” OR “chocolate cyst recurrence” OR “ovarian endometrioma recurrence” OR “endometriotic cyst recurrence”

5. side effects: “side effects” OR “adverse effects”

6. 1 AND 2 (245 results)

7. 2 AND 3 (980 results)

8. 1 AND 4 (50 results)

9. 3 AND 4 (293 results)

10. 1 AND 2 AND 5 (82 results)

11. 2 AND 3 AND 5 (179 results)
