# Supplementary material for: The Efficacy of Dienogest in Reducing Disease and Pain Recurrence After Endometriosis Surgery: a Systematic Review and Meta-Analysis
Source: Reprod Sci. 2023 May 22;30(11):3135–43. doi: 10.1007/s43032-023-01266-0 (PMC10643411; doi:10.1007/s43032-023-01266-0)
Supplement: Supplementary file 2 — ESM 2 [file 43032_2023_1266_MOESM2_ESM.docx]

| **Supplemental Table 1:** Risk of bias | | | | | | | | |
| --- | --- | --- | --- | --- | --- | --- | --- | --- |
|  |  |  |  |  |  |  |  |  |
| **Author and year** | **Risk of bias** | | | | | | | |
|  | Bias due to confounding | Bias in selection of partecipants into the study | Bias in classification of interventions | Bias due to deviations from intended interventions | Bias due to missing data | Bias in measurements of outcomes | Bias in selection of the reported result | Overall risk of bias |
| Adachi et al. 2016 | Low | Low | Low | Low | Low | Low | Moderate | Moderate |
| Lee et al. 2016 | Low | Low | Low | Low | Low | Serious | Low | Moderate |
| Ota et al. 2015 | Low | Low | Low | Low | Low | Low | Moderate | Moderate |
| Ouchi et al. 2014 | Low | Low | Low | Low | Low | Moderate | Moderate | Moderate |
| Yamanaka et al. 2017 | Low | Low | Low | Low | Low | Moderate | Low | Moderate |
|  |  |  |  |  |  |  |  |  |
