# Supplementary material for: The Efficacy of Dienogest in Reducing Disease and Pain Recurrence After Endometriosis Surgery: a Systematic Review and Meta-Analysis
Source: Reprod Sci. 2023 May 22;30(11):3135–43. doi: 10.1007/s43032-023-01266-0 (PMC10643411; doi:10.1007/s43032-023-01266-0)
Supplement: Supplementary file 3 — ESM 3 [file 43032_2023_1266_MOESM3_ESM.docx]

| **Supplemental Table 2:** List of excluded articles with reasons for exclusion | | |
| --- | --- | --- |
|  |  | |
| **Authors** | **Reason for exclusion** |  |
| Cosson et al. 2002 | Only pregnancy outcome is evaluated |  |
| Schindler et al. 2006 | No outcomes about pain, side effects or recurrence |  |
| Strowitzki et al. 2010 | No curative surgery (only diagnostic laparoscopy) |  |
| Hayashi et al. 2012 | No outcomes about pain, side effects or recurrence |  |
| Cucinella et al. 2013 | Postoperative administration of combined oral contraceptives |  |
| Yanase et al. 2014 | No outcomes about pain, side effects or recurrence |  |
| Angioni et al. 2015 | Non operated patients |  |
| Takenaka et al. 2015 | Dienogest used only before surgery |  |
| Vercellini et al. 2015 | Only 69% of patients have done surgery before treatment |  |
| Chandra et al. 2018 | No outcomes about pain, side effects or recurrence |  |
| Jeong et al. 2018 | A few patients have done surgery before treatment |  |
| Lee Ki H. et al. 2018 | Standard deviation is missing |  |
| Lee Sa Ra et al. 2018 | Standard deviation is missing |  |
| Romer et al. 2018 | Dienogest used only before surgery |  |
| Yu et al. 2018 | No outcomes about pain, side effects or recurrence |  |
| Techatraisak et al. 2019 | Only 87% of patients have done surgery before treatment |  |
| Kitajima et al. 2020 | No outcomes about pain, side effects or recurrence |  |
| Iwami et al. 2021 | Only pregnancy outcome is evaluated |  |
| Kim et al. 2021 | No outcomes about pain, side effects or recurrence |  |
| Dobrokhotova et al. 2021 | Standard deviation is missing |  |
|  |  | |
